# Development and internal validation of a multivariable nomogram for predicting postoperative complications and prolonged length of hospital stay in patients undergoing video-assisted thoracoscopic surgery: a single-center prospective cohort study

**DOI:** 10.3389/fmed.2026.1860903

**Published:** 2026-07-15

**Authors:** Yan Yang, Feng Shao, Guanghui Xia, Guanghong Wu, Jiefang Ding, Yang Yang

**Affiliations:** 1 Department of Thoracic Surgery, Nanjing Chest Hospital, Affiliated Brain Hospital of Nanjing Medical University, Nanjing, China; 2 Nursing Department, Nanjing Chest Hospital, Affiliated Brain Hospital of Nanjing Medical University, Nanjing, China

**Keywords:** complications, nomogram, prediction model, prolonged hospital stay, the concept of rapid rehabilitation surgery, thoracoscopic surgery

## Abstract

**Objective:**

To develop and internally validate a nomogram prediction model for postoperative complications and prolonged length of hospital stay in patients undergoing video-assisted thoracoscopic surgery based on the Enhanced Recovery After Surgery concept.

**Methods:**

We selected 399 lung cancer patients who underwent thoracoscopic surgery at the Thoracic Surgery Department of the Affiliated Brain Hospital of Nanjing Medical University from June 2022 to April 2023. Postoperative complications and length of stay were recorded. A nomogram prediction model was established using multivariate logistic regression analysis based on optimized features from the LASSO regression model. The model's diagnostic ability was evaluated using the ROC curve and C-index.

**Results:**

Multivariate logistic regression identified factors such as preoperative prediction, early rehabilitation nursing, and bed activity time as significant for postoperative complications and hospitalization time (*P* < 0.05). The nomogram prediction model highlighted abdominal breathing, exercise training, preoperative joint activities, and correct coughing as independent factors. The area under the ROC curve for predicting postoperative complications was 0.833 (95% CI: 0.784–0.936), with a C-index of 0.825. For prolonged hospital stay, the ROC area was 0.856 (95% CI: 0.749–0.941), with a C-index of 0.767.

**Conclusions:**

This model has high clinical prediction ability. Nursing interventions based on rapid rehabilitation surgery benefit lung cancer patients post-thoracoscopic surgery by reducing complications and shortening hospital stays. The prediction model, combined with basic clinical data, aids in creating better treatment plans.

## 1 Introduction

According to statistics, the incidence and mortality rates of lung cancer in China rank the highest (1, 2), and have shown a rapid growth trend in recent years. Uniportal thoracoscopic surgery, also abbreviated as “thoracoscopic surgery,” is the preferred treatment for lung cancer due to its advantages such as minimal surgical injury and rapid postoperative recovery of physiological function (3). However, studies have shown that patients undergoing thoracoscopic surgery are prone to complications, leading to prolonged hospitalization, which largely affects the prognosis (4).

Nursing interventions during the perioperative period, such as health education and psychological counseling, have been reported to play a positive role in promoting postoperative recovery and improving quality of life for patients (5). The concept of Enhanced Recovery After Surgery (ERAS) refers to evidence-based nursing practices validated for clinical application, aimed at alleviating patients' stress response during surgery. The advantages of ERAS include reducing complications and pain, promoting patient recovery, as well as shortening the length of hospital stay (6, 7). However, there have been no reports on predictive models for the effects of ERAS on postoperative complications and prolonged hospitalization in lung cancer patients undergoing thoracoscopic surgery.

Therefore, this study utilized clinical data from 399 cases of lung cancer undergoing thoracoscopic surgery admitted to the Affiliated Brain Hospital of Nanjing Medical University to explore high-risk factors for postoperative complications and prolonged hospitalization and to establish a nomogram predicting model, providing a basis for the prevention of postoperative complications and prolonged hospitalization in these patients.

## 2 Methods

### 2.1 Participants

This study was a prospective cohort investigation that enrolled 399 patients who underwent thoracoscopic surgery for lung cancer at the Department of Thoracic Surgery, the Affiliated Brain Hospital of Nanjing Medical University from June 2022 to April 2023.

Inclusion criteria were as follows: (1) diagnosis of lung cancer according to the diagnostic criteria outlined in the “Chinese Medical Association guideline for clinical diagnosis and treatment of lung cancer (2022 edition)” (8), and confirmed by clinical physicians; (2) undergoing thoracoscopic surgery; (3) age > 18 years; and (4) voluntary participation in this study with signed informed consent. Exclusion criteria included: (1) patients with metastatic lung cancer; (2) patients undergoing radiotherapy or chemotherapy; (3) patients participating in other clinical trials or studies. Elimination criteria included death during the trial or poor compliance.

This study was approved by the Ethics Committee of the Affiliated Brain Hospital of Nanjing Medical University (No. ZKX190469). All study subjects voluntarily participated and signed informed consent forms.

### 2.2 Sample size

Sample size calculation for prospective studies involves the formula:

$N={\left(\frac{Z_{1-\frac{\alpha }{2}\times \sigma }}{\delta }\right)}^{2}\;$

where *N* represents the sample size. According to literature, the incidence rate of complications in lung cancer patients is 38.60% (9). We determined that the enrollment of 358 patients would provide 80% power with the use of a permissible error δ of 1.0, a significance level α of 0.05 (two-sided), and assuming a dropout rate of 10%. Eventually, a total of 399 lung cancer patients were included in this study.

### 2.3 Study procedures

All study subjects underwent nursing interventions based on the principles of ERAS (10), with the specific procedures outlined as follows: (1) An ERAS nursing team, under the leadership of the head nurse, was established to develop comprehensive ERAS nursing protocols and conduct routine training sessions. All perioperative rehabilitation interventions were delivered and supervised by trained nursing staff within the ERAS team. No dedicated physiotherapists were involved in the routine implementation of the rehabilitation program during the study period. (2) Nursing staff provided health education to patients upon admission, ensuring their thorough understanding of the disease and explaining the risk factors associated with lung cancer and relevant precautions for thoracoscopic surgery. (3) Preoperative nursing guidance was provided to assist patients in mastering diaphragmatic breathing, pursed lip breathing, and proper coughing techniques. Enhanced communication was maintained with patients, addressing any preoperative concerns and providing psychological support and health education to help the patients cope positively with preoperative emotions. (4) Following postoperative consciousness recovery, pulmonary rehabilitation training was conducted based on the patient's condition, which included exercises such as diaphragmatic breathing, deep breathing, and active cycle of breathing techniques (ACBT), along with instruction on proper coughing techniques. In addition, standardized postoperative pain management was implemented according to institutional ERAS recommendations. Pain intensity was routinely assessed using the Numeric Rating Scale (NRS). Multimodal analgesia was adopted whenever possible, including non-steroidal anti-inflammatory drugs (NSAIDs), acetaminophen, and opioid analgesics when clinically indicated. The analgesic regimen was individualized according to pain severity and patient tolerance. Adequate pain control was considered an essential prerequisite for participation in respiratory rehabilitation and early mobilization activities. (5) A rehabilitation plan was devised for postoperative patients, encouraging early mobilization. Nursing staff assessed each patient's recovery status before mobilization and gradually increased the duration and intensity of activities according to individual tolerance and recovery progress. Early mobilization consisted of head-of-bed elevation, sitting upright in bed, bedside sitting, standing, walking exercises, and active joint movements. Mobilization was performed using standard hospital beds and conventional bedside chairs routinely available in the thoracic surgery ward. No specialized rehabilitation chairs or dedicated mobilization devices were used during the study period. The focus of the intervention was the timely initiation and progressive advancement of mobilization rather than the use of specific equipment. In addition, health education was provided to alleviate psychological stress and mitigate negative emotions during this period. (6) Before discharge, nursing staff provided patients with health education on post-discharge medication, lifestyle guidance, and other relevant instructions. Patients were given medication instructions for home use, informed about potential post-discharge situations, and provided with guidance to ensure postoperative recovery and improve quality of life after discharge.

### 2.4 Assessment indicators

(1) General information questionnaire: the questionnaire was designed by the research group and includes general demographic information of the study subjects such as age, gender, body mass index (BMI), smoking history, medical history.

(2) Health indicator survey: the questionnaire was compiled by the researchers which includes surgical time, intraoperative blood loss, Borg scale score, forced expiratory volume in 1 s (FEV1), maximum voluntary ventilation (MVV), and cough effectiveness score.

(3) Pain score: postoperative pain severity of the study subjects was assessed using the NRS, consisting of numbers from 0 to 10, where a higher number indicates more severe pain.

(4) Sleep quality assessment: The Pittsburgh Sleep Quality Index (PSQI) was used to evaluate the sleep quality of the study subjects. It comprises seven dimensions including subjective sleep quality and sleep latency, with a total score of 21 points. A higher score indicates poorer sleep quality.

(5) Postoperative complications: Patients were monitored throughout hospitalization for the occurrence of clinically relevant postoperative complications. Complications included pleural effusion requiring clinical observation or intervention, postoperative pneumothorax confirmed by chest imaging, atelectasis requiring respiratory therapy or additional clinical management, and postoperative delirium diagnosed according to standard clinical criteria by the attending physicians. The occurrence of at least one of these complications during hospitalization was classified as a postoperative complication event.

(6) Prolonged length of hospital stay (LOS): Hospital discharge was determined by the attending clinical team according to routine postoperative recovery criteria, including stable vital signs, adequate pain control, satisfactory pulmonary recovery, and absence of major complications requiring continued inpatient management. Postoperative LOS was calculated from the date of surgery to the date of discharge. Based on the distribution of LOS in the present cohort, the median postoperative LOS was 5 days. Therefore, LOS >5 days was defined as prolonged hospitalization, whereas LOS ≤ 5 days was considered non-prolonged hospitalization. This cutoff was selected to provide a clinically meaningful and data-driven classification consistent with previous studies evaluating postoperative recovery after thoracic surgery.

### 2.5 Statistical analysis

Data analysis was performed using SPSS 27.0 and *R* version 4.1.2. The Kolmogorov–Smirnov test was used to assess the normality of continuous variables. Normally distributed continuous variables were expressed as mean ± standard deviation (SD), whereas non-normally distributed variables were presented as median (interquartile range) [M(IQR)]. Categorical variables were described as frequency and percentage [*N* (%)].

The least absolute shrinkage and selection operator (LASSO) regression model was applied to identify the most informative ERAS-related variables and reduce potential multicollinearity among candidate predictors. Ten-fold cross-validation was performed to determine the optimal penalty parameter (λ), and variables with non-zero coefficients at the optimal λ value were retained for further analysis. These variables were subsequently entered into multivariable logistic regression models, and independent predictors were identified using a forward stepwise selection procedure based on statistical significance and model fit.

Based on the final multivariable logistic regression models, nomograms were constructed to estimate the probability of postoperative complications and prolonged length of hospital stay. Model discrimination was evaluated using receiver operating characteristic (ROC) curves, area under the curve (AUC), and concordance index (C-index). Because no independent external validation cohort was available, internal validation was performed using 1,000 bootstrap resamples to assess model stability, reduce the risk of overfitting, and obtain optimism-corrected performance estimates. Calibration and discrimination performance were subsequently evaluated using bootstrap-corrected estimates. A C-index >0.90 was considered indicative of excellent predictive performance, whereas values between 0.70 and 0.90 indicated acceptable to good discrimination. A two-sided *P*-value < 0.05 was considered statistically significant.

### 2.6 Quality control

Selection bias risk control: based on thorough literature review, a detailed and scientifically feasible research plan was formulated. Strict inclusion and exclusion criteria were applied to select study subjects. Personnel involved in the study underwent relevant training, followed by assessments to ensure qualification before participation in the study.

### 2.7 Information bias risk control

The data entry was performed using a double-entry method to ensure accuracy. Two researchers independently entered, checked, and analyzed clinical data. Questionnaires with response time less than 3 min or with the same option selected for more than 10 consecutive questions were excluded from the analysis

## 3 Results

### 3.1 Characteristics of study subjects

A total of 399 patients who underwent thoracoscopic surgery for lung cancer were included in this study. The mean age was 59.0±15.0 years, with a mean BMI of

23.8 ± 2.0 kg/m^2^. Smokers accounted for 23.8% of the cohort, with an average duration of 7.5±4.0 years. There were 179 (44.9%) male patients and 220 (55. 1%) female patients. Among all study subjects, 80 (29.6%) patients had concomitant hypertension, and 32 (11.9%) patients had diabetes mellitus. The mean duration of surgery for all study subjects was 1.4 (0.8) h, with an average blood loss of 45. 1 ± 13.7 ml. A total of 89 patients experienced postoperative complications, and a total of 156 patients had prolonged hospital stays (Table 1).

**Table 1:** General characteristics of study subjects (*n* = 399).

Variable	Median (interquartile range)/number (%)
Age, year	59.0 (15.0)
BMI, kg/m^2^	23.8 (2.0)
Smoking	7.5 (4.0)
Gender	
Male	179 (44.9%)
Female	220 (55. 1%)
Comorbidity	
Hypertension	80 (29.6%)
Diabetes mellitus	32 (11.9%)
Duration of surgery, h	1.4 (0.8)
Blood loss, ml	45.1 (13.7)
Postoperative complications	89 (22.3%)
Length of Hospital Stay, days	5.0(2.0)
Prolonged hospital stays	156 (39.2%)
Cardiovascular Disease	149(28.3%)
FEV1, L	2.3(0.7)
MVV, L/min	91.2(23.5)
Cough effectiveness	3.0(1.0)
Borg score (rest)	0.15(0.01)
Borg score (exercise)	1.0(0.3)
Surgery duration, hours	1.4(0.8)
PSQI score	5.2(2.7)
Pain assessment	2.0(1.3)

### 3.2 Duration of ERAS nursing and postoperative assessment indicators

Table 2 details the duration of ERAS - related interventions and postoperative rehabilitation for lung cancer patients undergoing thoracoscopic surgery. Preoperative rehabilitation includes modalities like diaphragmatic breathing (median 3.43 min, [3.0, 4.0] min) and health education (median 3.54 min, [3.0, 5.0] min). Postoperative pulmonary rehabilitation covers diaphragmatic breathing (median 3.36 min, [3.0, 4.0] min) and ACBT (median 3.49 min, [3.0, 4.0] min). Early bedside mobilization encompasses head - of - bed elevation (median 8.30 min, [5.0, 10.0] min), walking exercises (median 3.13 min, [3.0, 4.0] min), etc. These data reflect the time distribution of diverse rehabilitation measures in the ERAS pathway, providing a quantitative basis for evaluating the implementation of perioperative rehabilitation strategies and their potential impact on patient recovery.

**Table 2:** Duration of ERAS and postoperative rehabilitation effects in patients with thoracoscopic surgery for lung cancer.

Variable	Classification	Median	[P25, P75]
Preoperative rehabilitation, minutes	Diaphragmatic breathing	3.43	[3.0, 4.0]
	Pursed lip breathing	3.19	[3.0, 4.0]
	Coughing training	3.07	[3.0, 4.0]
	Exercise training	3.01	[3.0, 4.0]
	Psychological support	3.10	[3.0, 4.0]
	Health education	3.54	[3.0, 5.0]
Postoperative pulmonary rehabilitation, minutes	Diaphragmatic breathing	3.36	[3.0, 4.0]
	Deep breathing	3.21	[3.0, 4.0]
	ACBT, minutes	3.49	[3.0, 4.0]
Early bedside mobilization, minutes	Correct Coughing	3.15	[3.0, 4.0]
	Head of Bed Elevation	8.30	[5.0, 10.0]
	Sitting Position	3.92	[3.0, 5.0]
	Bedside Sitting	3.45	[3.0, 5.0]
	Walking Exercises	3.13	[3.0, 4.0]
	Active joint movements	2.84	[2.0, 3.0]
	Health education	3.51	[3.0, 4.0]

ACBT, active cycle of breathing techniques; ERAS, enhanced recovery after surgery.

### 3.3 Development and validation of predictive model of the impact of ERAS on postoperative recovery in patients with lung cancer undergoing thoracoscopic surgery

#### 3.3.1 Factors associated with postoperative complications in patients with lung cancer undergoing thoracoscopic surgery

Multivariable logistic regression analysis revealed that preoperative rehabilitation (diaphragmatic breathing, psychological support, and health education), postoperative pulmonary rehabilitation (diaphragmatic breathing, ACBT, and correct coughing), and early bedside activities after surgery (raising the head of the bed, sitting, bedside sitting, and health education) were independent factors influencing postoperative complications in patients undergoing thoracoscopic surgery (*P* < 0.05, Table 3).

**Table 3:** Multivariable logistic regression analysis of risk factors for postoperative complications in patients after thoracoscopic surgery.

Variable		β	SE	*P*	OR	95% CI	
						LB	UB
**Preoperative rehabilitation, minutes**	Diaphragmatic Breathing	0.144	0.251	0.038	1.732	1.059	2.834
	Pursed Lip Breathing	0.247	0.368	0.073	1.172	0.569	2.411
	Coughing Training	0.306	0.303	0.088	1.105	0.610	2.001
	Exercise Training	0.130	0.146	0.077	1.317	0.989	1.753
	Psychological Support	0.462	0.197	0.001	2.381	1.619	3.503
	Health Education	0.056	0.176	0.041	1.418	1.004	2.003
**Postoperative pulmonary Rehabilitation, minutes**	Diaphragmatic Breathing	0.061	0.182	0.049	1.594	1.116	2.275
	Deep Breathing	0.009	0.229	0.064	1.514	0.967	2.371
	ACBT	0.017	0.106	0.034	1.475	1.199	1.815
	Correct Coughing	0.058	0.115	0.041	1.590	1.270	1.990
**Early bedside mobilization, minutes**	Head of Bed Elevation	0.009	0.021	0.046	1.487	1.427	1.550
	Sitting Position	0.031	0.103	0.012	1.547	1.263	1.893
	Bedside Sitting	0.008	0.141	0.040	1.512	1.146	1.994
	Walking Exercises	0.177	0.131	0.051	1.256	0.971	1.625
	Active Joint Movements	0.092	0.178	0.064	1.368	0.965	1.938
	Health Education	0.110	0.128	0.026	1.675	1.303	2.154

*P* < 0.05 indicates statistical significance. The covariables in the model include age, gender, BMI, duration of smoking, concomitant cardiovascular disease, duration of surgery, volume of intraoperative bleeding.

**Figure 1:**
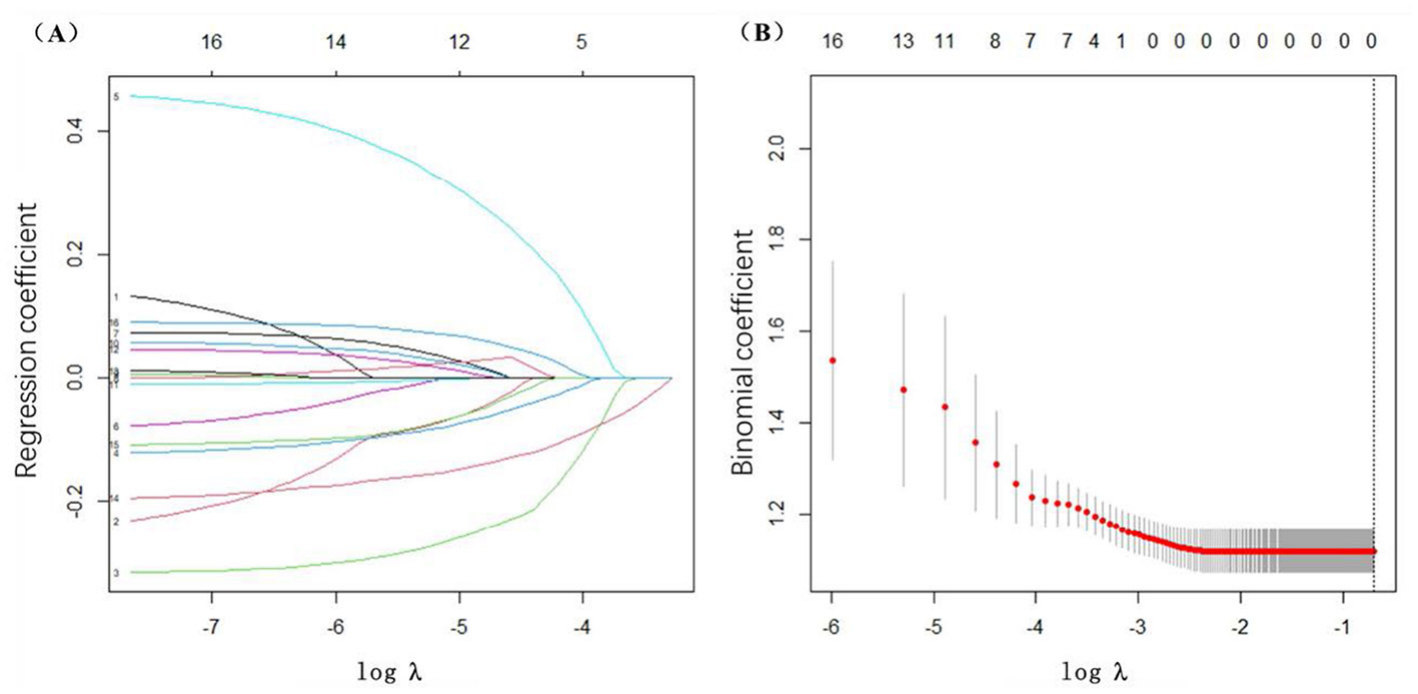
Texture feature selection using the LASSO binary logistic regression model for postoperative complications. **(A)** LASSO coefficient profiles (λ value); **(B)** Tuning parameter (λ) selection in the LASSO model used 10-fold cross-validation via minimum criteria.

**Figure 2:**
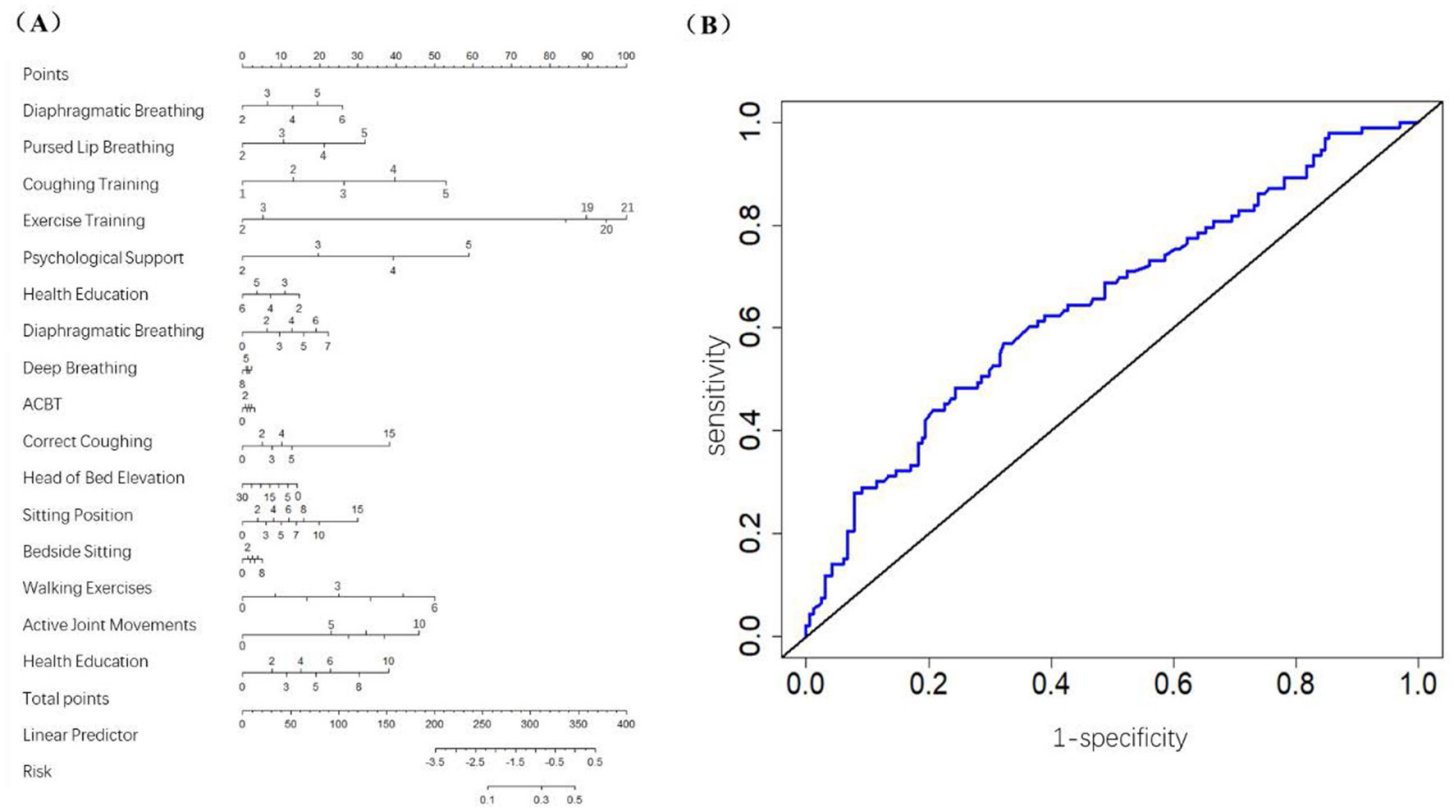
Risk prediction nomogram for postoperative complications and ROC.

The nomogram indicates that the major contributors for postoperative complications in thoracoscopic surgery are diaphragmatic breathing, preoperative exercise training, active joint exercises, and correct coughing (Figure 1). The area under the ROC curve (AUC) was 0.833 (95% CI: 0.784–0.936), suggesting that the model has relatively high clinical predictive ability (Figure 2). Internal validation using 1,000 bootstrap resamples yielded a corrected C-index of 0.825, indicating good discriminative performance and model stability.

#### 3.3.2 Factors associated with prolonged hospital stay in patients with lung cancer undergoing thoracoscopic surgery

Multivariable logistic regression analysis revealed that preoperative rehabilitation (coughing training, exercise training, psychological support, and health education), postoperative pulmonary rehabilitation (diaphragmatic breathing, ACBT), and early bedside activities after surgery (raising the head of the bed, sitting, bedside sitting, walking exercises, and active joint movements) were independent factors influencing prolonged hospital stay in patients undergoing thoracoscopic surgery (*P* < 0.05, Table 4).

**Table 4:** Multivariable logistic regression analysis of risk factors for prolonged hospital stay in patients after thoracoscopic surgery.

Variable		β	SE	*P*	OR	95% CI	
						LB	UB
**Preoperative rehabilitation, minutes**	Diaphragmatic breathing	0.232	0.221	0.089	1.349	0.874	2.080
	Pursed lip breathing	0.036	0.293	0.090	1.762	0.993	3.128
	Coughing training	0.035	0.251	0.030^*^	1.642	1.003	2.687
	Exercise training	0.101	0.100	0.031^*^	1.660	1.364	2.020
	Psychological support	0.283	0.174	0.010^*^	1.991	1.417	2.798
	Health education	0.058	0.145	0.027^*^	1.590	1.196	2.112
**Postoperative Pulmonary**	Diaphragmatic breathing	0.224	0.166	0.018^*^	1.877	1.355	2.601
**Rehabilitation, minutes**	Deep breathing	0.090	0.211	0.067	1.371	0.907	2.072
	ACBT	0.124	0.095	0.019^*^	1.324	1.100	1.594
	Correct coughing	0.178	0.134	0.083	1.255	0.965	1.631
**Earlybedside mobilization, minutes**	Head of bed elevation	0.009	0.017	0.017^*^	1.486	1.437	1.538
	Sitting position	0.121	0.085	0.015^*^	1.694	1.433	2.002
	Bedside sitting	0.145	0.128	0.026^*^	1.298	1.010	1.668
	Walking exercises	0.107	0.113	0.035^*^	1.669	1.336	2.084
	Active joint movements	0.043	0.148	0.011^*^	1.437	1.075	1.921
	Health education	0.193	0.119	0.077	1.237	0.980	1.563

*P* < 0.05 indicates statistical significance. The covariables in the model include age, gender, BMI, duration of smoking, concomitant cardiovascular disease, duration of surgery, volume of intraoperative bleeding.

**Figure 3:**
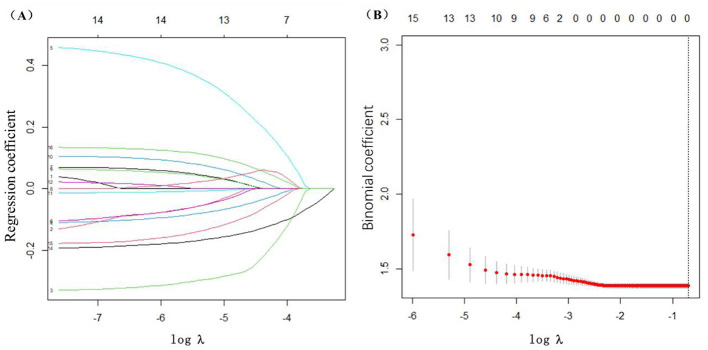
Texture feature selection using the LASSO binary logistic regression model for prolonged hospital stay. **(A)** LASSO coefficient profiles (λ value); **(B)** Tuning parameter (λ) selection in the LASSO model used 10-fold cross-validation via minimum criteria.

**Figure 4:**
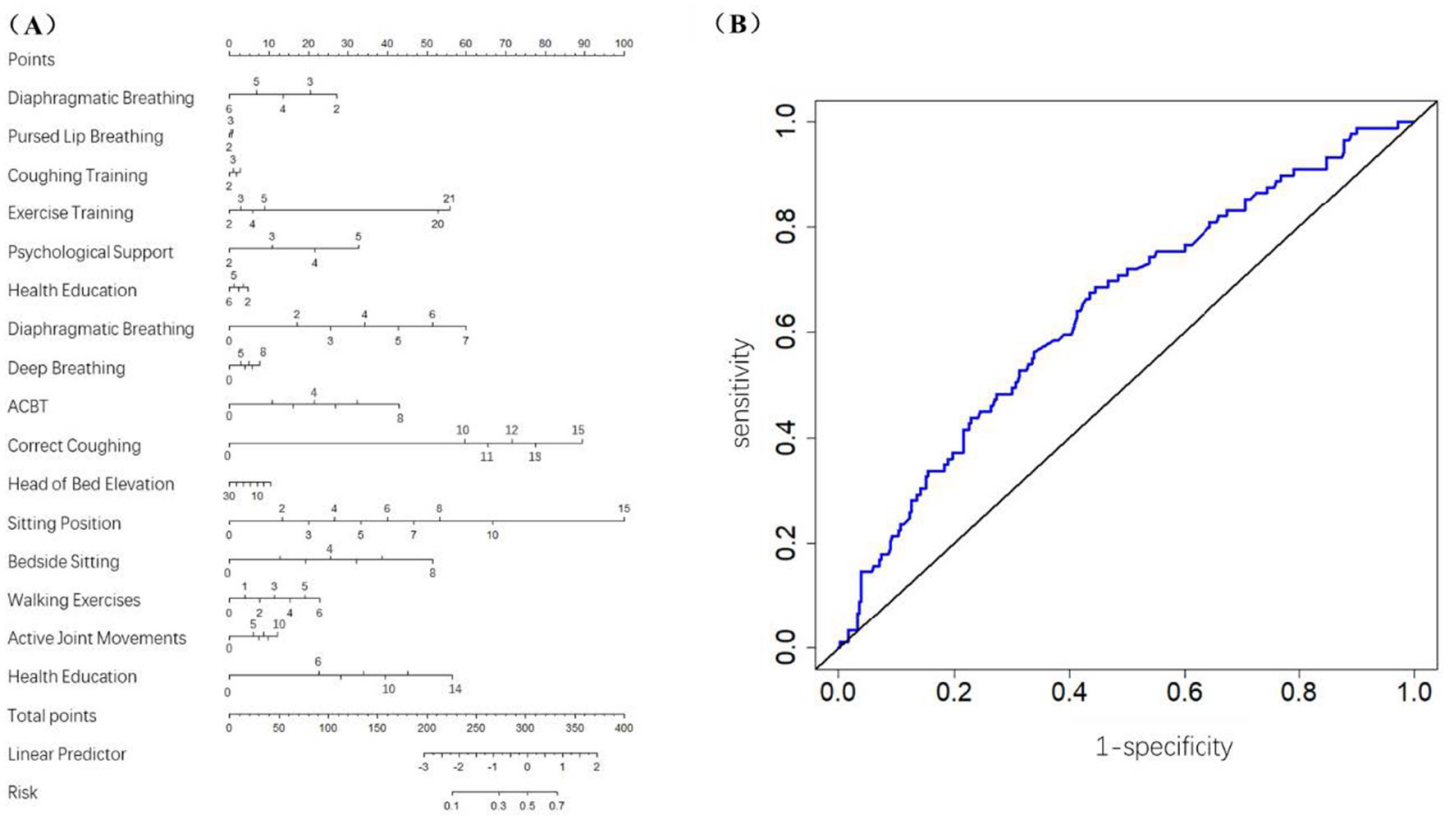
Risk prediction nomogram for prolonged hospital stay and ROC.

The nomogram indicates that the major contributors for postoperative complications in thoracoscopic surgery are deep breathing, diaphragmatic breathing, and correct coughing (Figure 3). The area under the ROC curve (AUC) was 0.856 (95% CI : 0.749 ~ 0.941)), suggesting that the model has relatively high clinical predictive ability (Figure 4). Internal validation using 1,000 bootstrap resamples yielded a corrected C-index of 0.767, indicating acceptable discriminative ability of the model.

## 4 Discussion

Currently, thoracoscopic surgery is commonly used in clinical practice for treating lung cancer patients, which can remove the primary lesion and improve patient survival rates (11). Due to the minimally invasive nature of thoracoscopic surgery and the various factors affecting surgery, patients may experience delayed recovery of vital signs and prolonged postoperative hospital stays. Therefore, during surgical treatment, scientific and effective nursing interventions can promote postoperative recovery, shorten hospital stays, reduce complications, and improve quality of life, which is of great significance for patient recovery. ERAS is an intervention that utilizes effective nursing measures to reduce surgical stress responses, lower postoperative complications, and enhance overall nursing effects during the perioperative period (12). Preoperatively, patients receive health education on their disease and lifestyle habits, enabling them to have a better understanding of their condition before treatment. Additionally, through communication, nurses can assess the preoperative psychological status of patients and provide timely psychological counseling.

Our study found that preoperative rehabilitation, postoperative lung rehabilitation nursing, and early bedside activities were all factors associated with postoperative complications and prolonged hospital stays after thoracoscopic surgery. This has also been confirmed by other studies (13, 14). The reason may be that preoperative rehabilitation for patients exercises their cardiopulmonary function to some extent, thereby reducing the risk of postoperative complications and prolonged hospital stays. Postoperative lung rehabilitation and early bedside activities effectively remove secretions accumulated in the lungs, reducing the risk of bacterial invasion and subsequent complications such as postoperative pulmonary infection. By implementing interventions based on the concept of ERAS for patients with lung cancer, preoperative rehabilitation is conducted by nursing staff upon admission, including diaphragmatic breathing training, lip closure breathing, cough training, exercise training, psychological support, and health education. This helps patients to have a proper understanding of their disease and thoracoscopic surgery, further eliminating negative emotions. Postoperatively, nursing is primarily conducted through lung rehabilitation and early bedside activities, which promote the recovery of vital signs such as heart rate, blood pressure, and respiration after thoracoscopic surgery, reduce the incidence of complications, and shorten postoperative hospital stays. This study established two nomogram prediction models, with respective areas under the ROC of 0.833 (95% CI: 0.784–0.936) and 0.856 (95% CI: 0.749–0.941), indicating that these models constructed in this study have high clinical predictive ability. This suggests that ERAS can help reduce the incidence of postoperative complications and shorten postoperative hospital stays, contributing to the recovery of patients' condition. Compared with previous studies investigating ERAS protocols in thoracic surgery, the present study provides several novel findings. Most previous studies primarily focused on evaluating the overall effectiveness of ERAS pathways on postoperative outcomes, such as reducing complications, shortening hospital stay, and improving recovery. However, relatively few studies have attempted to quantify the individual contribution of specific perioperative rehabilitation measures or to develop practical prediction tools that can identify patients at increased risk of adverse postoperative outcomes. In the current study, we not only confirmed the beneficial effects of ERAS-related interventions but also incorporated multiple perioperative rehabilitation variables into a nomogram-based prediction model. This approach enables individualized risk assessment and may facilitate early identification of patients who require intensified perioperative support and rehabilitation. Such personalized management strategies are increasingly emphasized in modern thoracic surgery and perioperative medicine. Furthermore, the predictive models demonstrated satisfactory discrimination, suggesting potential utility in routine clinical practice. By integrating readily obtainable clinical and nursing-related variables, the nomograms may assist clinicians and nursing teams in optimizing resource allocation, tailoring rehabilitation programs, and implementing targeted interventions for high-risk patients. Therefore, beyond confirming the effectiveness of ERAS, this study contributes a practical risk stratification tool that may support precision perioperative care in patients undergoing thoracoscopic surgery.

Respiratory function exercise is crucial for lung cancer patients, and good lung function is the foundation for postoperative recovery (15, 16). Studies have shown that targeted respiratory function training under the concept of ERAS can effectively exercise the respiratory muscles in the short term, playing a more positive role in the postoperative recovery of patients undergoing thoracoscopic surgery (17). Diaphragmatic breathing and deep breathing training can increase tidal volume, respiratory efficiency, arterial oxygen pressure, alleviate dyspnea, and improve lung ventilation function. ACBT is a flexible, self-controlled flexible therapy and respiratory rehabilitation training model that effectively clears bronchial secretions and improves lung function without exacerbating hypoxemia and airflow resistance. Postoperative pain may also influence the effectiveness of rehabilitation interventions. Inadequate pain control may reduce patients' willingness to participate in respiratory exercises, effective coughing, and early mobilization, thereby limiting pulmonary recovery and delaying functional improvement. Consequently, poor pain control may indirectly increase the risk of postoperative complications and prolonged hospitalization. Although pain scores were assessed in the present study, the relationship between pain severity, rehabilitation adherence, and clinical outcomes was not specifically analyzed and warrants further investigation.

Studies suggested that early bedside activities can effectively promote the recovery of patients' postoperative physiological functions, reduce the risk of thrombosis caused by prolonged bed rest, and reduce the risk of decreased mobility (18). Notably, early mobilization in the present study was achieved using standard ward facilities, including conventional hospital beds and bedside chairs, without specialized rehabilitation equipment. This finding suggests that the clinical benefits of early mobilization may be achievable in routine thoracic surgery practice without substantial additional resource investment, thereby supporting the feasibility and scalability of ERAS implementation. Furthermore, early bedside activities can promote the recovery of gastrointestinal function, accelerate the absorption of nutrients, and reduce the incidence of postoperative gastrointestinal adverse reactions (19–21).

This study has several limitations. The survey was conducted only in a single center, which may introduction certain bias. There are numerous factors that could influence complications and prolonged hospital stay, and patients themselves exhibit considerable heterogeneity. Additionally, although this study included prospectively collected data, no independent external validation cohort was available. Therefore, the nomogram was validated internally using bootstrap resampling only. Further external validation in independent, large-scale, multicenter cohorts is needed before broader clinical application. Subsequent efforts will focus on clinical treatment using the principles of ERAS. However, the introduction of ERAS in thoracoscopic surgery for lung cancer patients

has shown significant effects on improving vital signs, reducing complications, shortening hospital stays, and enhancing the quality of clinical care, thereby providing assurance for postoperative recovery of these patients. This study has positive implications and clinical value for improving patient quality of life. The results from this study can serve as a reference for adjusting and implementing corresponding diagnostic and nursing strategies, reducing the occurrence of postoperative complications, and more effectively promoting postoperative recovery.

## Data Availability

The original contributions presented in the study are included in the article/supplementary material, further inquiries can be directed to the corresponding author.
